# Nursing 12-Hour Shifts and Patient Incidents in Mental Health and Community Hospitals: A Longitudinal Study Using Routinely Collected Data

**DOI:** 10.1155/2023/6626585

**Published:** 2023-09-06

**Authors:** Chiara Dall'Ora, Ourega-Zoé Ejebu, Jeremy Jones, Peter Griffiths

**Affiliations:** ^1^School of Health Sciences, University of Southampton, Southampton SO17 1BJ, UK; ^2^NIHR Applied Research Collaboration Wessex, Southampton SO16 7NP, UK

## Abstract

Shifts of 12 hours or longer are common in nursing services within general hospital wards. Concerns have been raised about their safety, but previous research has mostly used staff-reported measures of quality and safety and has occurred in general hospital settings only. This study aims to measure the association between the use of 12+ hour shifts in nursing staff (including registered nurses, healthcare support workers or nursing assistants, and nursing associates) and the rate of patient incidents in mental health and community hospitals. This is a longitudinal study using routinely collected data from two mental health and community NHS trusts in the South of England. We accessed rosters of nursing staff and patient incident data from April 2018 to March 2021. We extracted 1,018,971 shifts and excluded those not worked by nursing staff, with a final sample of 898,143 shifts. We extracted 53,078 incidents. We only included incidents that involved patients and that occurred in wards. Our final sample consisted of 38,373 patient incidents. We linked all patient incidents and nurses' worked shifts at the ward-day level. Depending on the distribution of incident rates, we used either negative binomial mixed-effects models or Poisson mixed-effect models to investigate the association between the proportion of 12+ hour shifts and all patient incidents, violence against staff, falls, self-injury, disruptive behaviour, and medication management incidents at the ward-day level. We found a relationship between 12+ hour shifts and the incident rate. Compared to days in wards with no long shifts, increasing the proportion of long shifts was initially associated with a small increase in the overall rate of incidents, but the rate increased sharply as the proportion of long shifts was above 70%. Rates of self-injury increased more steadily as the proportion of long shifts increased. The mandatory implementation of long shifts should be discouraged.

## 1. Introduction

Despite concerns about its implications for patient safety and quality of care, shift work in hospital nursing remains a reality because healthcare needs to be provided 24-hour a day. Concerns arise due to shift work causing circadian disruptions among workers, leading them to experience fatigue and lower alertness and vigilance [1–4]. In a sector where decreased staff vigilance and monitoring have major implications for patient safety, [5, 6] and where workforce shortages are increasingly high [7], healthcare managers face the challenge of planning shifts in a way that is both safe and efficient.

Shift work can be organised in a variety of ways [8, 9]. However, in many countries, including in England's NHS inpatient general hospital settings, long shifts of 12 hours or longer have become the norm for nurses [10–12]. When long shifts were first introduced, the assumption was that they would save money and allow deploying the workforce more efficiently by reducing overlaps between shifts while increasing quality and safety of care. This was due to the belief that by scheduling two 12+ hour shifts instead of three 8-hour shifts, patients would benefit from increased continuity of care across 24 hours [13–15]. This shift pattern would also lead to reduced handovers, which are critical information-passing moments where miscommunication can occur and has the potential to damage patient care [16].

After their introduction in the late 70s' in the US, 12+ hour shifts increased steadily in the UK and several other countries in Europe, but there was little if any robust evaluation of their impact [17]. It is only recently that more rigorous studies using large samples and objective roster data have started to shed light on the effect of long shifts [18]. The overall emerging picture points to a negative effect on nurses' health and well-being [19], including sickness absence [10, 11], burnout [20, 21], and intention to leave their job [22]. Far from enabling staff to perform more productively, these long shifts have also been associated with higher rates of errors and patient safety-related outcomes [12, 23, 24]. The hypothetical link between 12+ hour shifts and jeopardised patient safety is the inshift fatigue increase [2]. Since fatigue during the shift increases exponentially after the first 8 hours and accumulates over consecutive shifts [4, 25], and fatigue is linked to accidents and performance impairments [26], the consequences for patient safety could be serious. Nonetheless, most evidence around the safety of 12+ hour shifts is largely based on self-reported measures derived from surveying nurses [18]. Such evidence should not be entirely discounted, as some studies demonstrated the correlation between staff ratings of quality and safety and objective indicators [27]. However, the common method variance bias associated with subjective measures remains [28].

With the increasing availability of routinely collected data for research purposes, studies relating objective work hours to patient incidents have started to emerge. However, all these studies have considerable limitations. In one instance, incident data were aggregated at the hospital level and related to the hospital's most typical shift length [29]. The average shift length at the hospital level gives little indication of what happens to patient incidents on a daily basis, at a ward level, depending on shift patterns nurses work. Another small-sample study used objective data and focused on the number of shifts worked prior to a patient experiencing hypoglycemia [30]. However, it did not consider patient acuity or any patient characteristics in their analysis. While the association is plausible, other mechanisms due to patient acuity/characteristics cannot be discounted. A further study considered the number of staff working overtime per shift in relation to seclusion incidents in a forensic setting, but no data on staff total worked hours per shift were available [31]. Another study focused on compliance with vital sign observation protocols. It found that when healthcare assistants were working 12+ hour shifts, vital sign observations were more likely to be delayed [32]. However, a delay in completing and recording vital sign observations did not necessarily lead to incidents.

In addition, the available evidence comes from general acute care hospitals, but the effects of shift length are likely to be context-specific, and neither the uptake of 12+ hour shifts nor the impact on outcomes is known in mental health and community inpatient hospitals. Therefore, our study aimed to measure the association between the use of 12+ hour shifts on each ward-day and the rate of patient incidents in mental health and community settings.

## 2. Methods

This was a longitudinal study using routinely collected data from two large community and mental healthcare providers in the South of England. Community hospitals in this context are smaller hospitals that do not offer acute inpatient care or emergency services. Inpatient services in these hospitals support the rehabilitation and recovery of patients, who are often admitted after being treated in general acute care hospitals for acute episodes. The two trusts comprise 23 hospitals and sites spanning across a wide geographical area providing care to more than two million people. Because our main variable of interest was shift patterns, we focused on the 49 wards that provided inpatient care. We obtained NHS Health Research Authority approval (20/HRA/3881) and ethical approval from the University of Southampton Ethics Committee (Approval ID: 57489.A4).

We related repeated measures of shift and staffing configurations from the 49 wards and the number and type of incidents occurring on those wards on the same day. We retrospectively analysed our data for three years in total; we extracted shift patterns worked by nursing staff from the trust electronic rostering systems' from April 2018 to March 2021. By nursing staff or “nurses,” we mean registered nurses (who completed a training programme approved by the Nursing and Midwifery Council, usually a three- to four-year university degree), nursing assistants (also known as healthcare support workers/healthcare assistants, who assist with hygiene, feeding, and other aspects of fundamental nursing care), and nursing associates (staff who completed a formal two-year diploma and help bridge the gap between registered nurses and assistants/support workers). In total, we extracted 1,018,971 shifts. We then selected shifts worked in inpatient wards only, and we excluded all shifts that were not worked due to sickness absence and any other leave. This resulted in a sample of 898,143 shifts.

Patient incident data were derived from the trust incident reporting system from April 2018 to March 2021, for a total of 38,373 incidents. We only included incidents that involved patients and that occurred in wards. We excluded any incidents that involved staff only or occurred outside of the ward, for example, in the car park or during patient transfer to other facilities. In the patient incident reporting system, each incident was labelled to describe its impact: no harm, low/minimal harm, moderate harm, and major harm, and we retained this information for analysis. Patient incidents had no patient identifiers, including demographics, attached to them. We collected the number of occupied beds for each day and ward from the patient admission dataset using the midnight census. By the midnight census, we mean the number of beds in each ward that are occupied at midnight.

Our primary outcome was the total number of patient incidents per ward day. We also calculated the number of incidents with any harm. We then focused on the five most recurring incidents separately: violence against staff, falls, self-injury, disruptive behaviour, and medication management incidents. We calculated the total number of each respective incident per ward day. To account for different ward sizes, when reporting the volume of incidents, we calculated incident rates. Incident rates were calculated as the number of incidents per 1000 bed days.

Our main variable of interest was the proportion of 12+ hour shifts in each ward day. We also calculated staffing levels by dividing the total number of nursing staff by the number of occupied beds at midnight, the proportion of shifts worked by bank/agency nurses, and the proportion of shifts worked by registered nurses. All these variables were calculated at the ward-day level, and ward day was the unit of analysis.

We first conducted a descriptive analysis to determine the frequency of incidents, overall and at the ward level, to identify the five most frequently recurring incidents. We also described the distribution of 12+ hour shifts across each trust and by ward. For both incidents and shift patterns, we checked the distribution across years.

We measured the association between the proportion of long shifts and the number of patient incidents with negative binomial mixed-effects models, with the number of occupied beds as the offset. We then modelled the association between the proportion of long shifts and the number of incidents with any harm and for the most frequently occurring patient incidents (i.e., violence against staff, falls, self-injury, disruptive behaviour, and medication management incidents). Due to the absence of overdispersion (i.e., when accounting for all predictors, the variance equalled the mean), some models used Poisson mixed-effects models.

As previous research found nonlinear associations between shift patterns and outcomes [11, 33], we added quadratic and cubic terms to the models to model nonlinear effects. We used the Akaike information criterion (AIC) and Bayesian information criterion (BIC) to compare fit between models, preferring models with lower AIC/BIC. In all models, we controlled for the proportion of shifts worked by bank/agency, the proportion of shifts worked by registered nurses, and staffing levels, because these variables have been previously associated with variation inpatient outcomes [7, 34]. We also controlled for setting (physical health ward, adult mental health, and child mental health) because some incidents were more relevant to mental health settings and because incident rates were higher in children's wards. All analyses included ward as a random effect and were at the ward-day level. We checked the variance inflation factor (VIF) to ensure there was no or little (i.e., VIF <5) multicollinearity. Analyses were conducted in R 4.1.3 [35] using package lme4 [36].

## 3. Results

Our sample consists of 50,499 ward days. There were 38,373 patient incidents (25% incurring in no harm) occurring over 19,074 ward days, while there were no incidents in the remaining 31,425 ward days. The overall incident rate was 55.72 incidents per 1000 occupied bed days. Considering figures published at the time of the study, this is in line with the average incident rate across England in mental health and community trusts (i.e., 57.26 incidents per 1000 occupied bed days) [37]. The incident rate was higher in mental health wards (74.62 incidents per 1000 occupied bed days) than in physical health wards (22.93 incidents per 1000 occupied bed days). The incident rate increased every year, with 46.88 incidents per 1000 bed days in year 1 (2018-19), 54.26 in year 2 (2019-20), and 68.16 in year 3 (2020-21).

There was considerable variation in the distribution of 12+ hour shifts. On average, in each ward day, 26.5% of the shifts were long shifts, and the median was 20%. There were 8,543 (17%) ward days with no long shifts, and most ward days (*n* = 10,154, 20%) used between 1 and 10% of long shifts. On 578 ward days (1%), there were only long shifts.

The use of 12+ hour shifts varied between and within wards, as shown in the box plot in Supplementary Material Figure 1.

**Figure 1:**
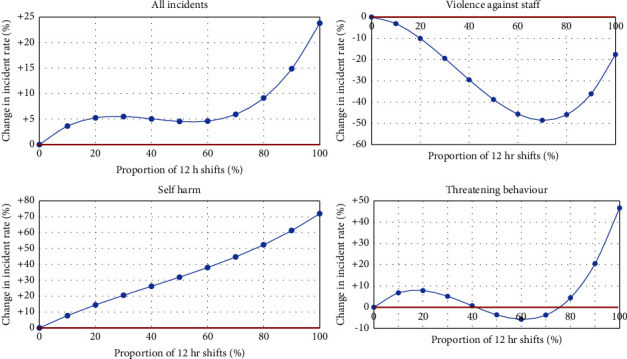
Risks of incidents and use of 12+ hour shifts.

The use of long shifts varied across mental and physical health wards (Figure 1). Shifts of 12+ hours were used more frequently in mental health children wards (mean: 60%) and physical health (mean 37%) than in mental health wards (mean: 18%). The use of 12+ hour shifts at the ward-day level increased across years. Specifically, in year 1, on average, long shifts represented 15.4% of all shifts, 20% in year 2, and 25% in year 3.

The mean proportion of shifts worked by substantive staff was 78%, while 19% and 3% of shifts were worked by bank and agency staff, respectively. The mean nursing staff-per-bed ratio was 1.52. The mean proportion of hours covered by RNs was 41%, and 58% were worked by healthcare support workers and nursing associates.

Table 1 reports the unadjusted and fully adjusted estimates for the long shift linear, squared, and cubic terms from the negative binomial and Poisson mixed-effects regressions. The full models including the covariates are available in Supplementary Material (Table S1).

We found a statistically significant association between the proportion of long shifts and patient incidents across both adjusted and unadjusted regression models. Focusing on specific incident types, we found statistically significant associations for violence against staff, self-injury, and disruptive behaviour.

Because nonlinear effects are difficult to convey based on point estimates only, we produced graphical representations from the B coefficient of the cubic term of 12-hour shifts, focusing on outcomes where one or more of the 12+ hour shift terms was statistically significant (Figure 1). We categorised the proportion of 12+ hour shifts in 10% bands.

For all outcomes, we found that high proportions (i.e., 70% or above) of 12+ hour shifts are associated with higher incident rates. For self-injury, there was evidence of a linear relationship, while for patient incidents and disruptive behaviour, the relationship was nonlinear with substantial increases only happening with high proportions (i.e., 80% or above) of 12+ hour shifts. For violence against staff, there was a different relationship, with the risk decreasing as the proportion of long shifts increases up to 80%. The lowest risk of such incidents occurs when between 60 and 70% long shifts are used at the ward-day level. Then, the relationship reverts: the risk of violence against staff increases exponentially between 80 and 100% but remains lower in comparison to ward days when no long shifts are used.

We conducted additional sensitivity analyses. Since testing for interactions between the proportion of long shifts as cubic terms and setting meant the model did not converge, we did a sensitivity analysis with children and mental health settings only, and we found that the relationships did not differ from those derived from models with three settings in.

Since the incident rate of violence against staff was considerably higher in one ward (i.e., mean incident rate = 409 vs. mean incident rate across other wards = 18), likely due to different reporting practices and a patient population substantially different from other wards, we excluded this ward for total incidents and violence against staff. Adverse effects of very high and very low proportions of 12-hour shifts were eliminated in the sensitivity analysis. B coefficients and standard errors from the negative binomial mixed-effects models and a graph to display associations are attached in Supplementary Material (Table S2 and Figure S2).

## 4. Discussion

To our knowledge, this was the first longitudinal study using nursing shift and patient incident data derived from hospital systems to measure the association between the use of long shifts and the risk rate of patient incidents in mental health and community inpatient settings. After controlling for relevant variables that could influence the patient incident rate, we found that using long shifts at a ward-day level is significantly associated with the rate of patient incidents. Variation in the use of long shifts was also associated with three specific incident types, namely, violence against staff, self-injury, and patients displaying disruptive behaviour. We did not find statistically significant associations for falls or medicines management incidents.

Higher proportions of long shifts were associated with higher incident rates for all incident types, with the highest risk when 12+ hour shifts represented 70–100% of the shifts on a ward day. While previous studies found that the association between long shifts and other outcomes is not linear [11, 33], these nonlinear relationships with incidents linked to the daily proportion of 12+ hour shifts on a ward were not observed before. It is nonetheless consistent with the hypothesised mechanism that high proportions of long shifts lead to higher staff fatigue, and this in turn leads to higher patient incident rates [2, 25].

For two incident types (i.e., violence against staff and disruptive behaviour), we found that a proportion of between 20 and 70% of long shifts was associated with a lower incident rate. These mixed patterns may permit staff to work the shift of their choice, where those most able to tolerate long shifts choose to work this shift pattern, adapting to it. Nonetheless, the adverse effects of long shifts cannot be eliminated, and we found negative consequences on patient safety when all shifts in a ward are 12 hours or longer. This finding has also been observed in relation to sickness absence when entire wards were moved to 12+ hour shifts [10]; however, it has never been observed in relation to patient incidents. While the degree of choice and self-selection might be likely mechanisms underlying the association between long shifts and some patient incidents, our current data do not allow us to test this.

Our results indicate that associations for different outcomes differ, suggesting that aggregating all incidents together might have hidden diverse effects. Using high proportions of long shifts at a ward-day level was also associated with an increase in the risk of disruptive behaviour from patients. While patient acuity is likely to be the main determinant of these types of incidents, decreased staff vigilance due to higher fatigue resulting from working long shifts [4, 25] might also play a role. Future studies that indirectly monitor inshift fatigue could shed light into this hypothesised mechanism [38].

### 4.1. Limitations

Across years, both the incident rate and the proportion of long shifts increased, meaning that it is impossible to rule out that any association between incidents and the proportion of long shifts is simply a result of changes in incident rates over time.

A further limitation is that the study was conducted in two trusts. Although these were large trusts made up of several hospitals dispersed over a wide geographical area in England, the results might not generalise to other hospitals in other geographical locations or other inpatient settings. In addition, we included diverse patient populations, which influenced the variation in incident rates, although our sensitivity analyses reassure us that the association between 12+ hour shifts was not influenced by the ward setting and underlying patient population.

In addition, there is much debate in the literature around the appropriateness of using incident reporting data due to dubious quality in the reporting and variation in staff attitudes towards incident reporting [39, 40]. While this limitation cannot be easily addressed, and the real incident rate is difficult to estimate, incident reports from hospital systems remain the most widely used measure of patient safety when using routinely collected data. In recent years, incident reporting has become standard practice in many healthcare settings [41]. Moreover, we did not collect any data relating to the nursing staff's experiences of shift work and quality of work as perceived by them, including whether they had access to any hospital-wide interventions to support their fatigue and psychological wellbeing. There is evidence that such interventions can improve workforce wellbeing and performance [42].

## 5. Conclusions

The consequences of patient incidents such as self-injury and disruptive behaviour are serious [43], and using high proportions of long shifts is associated with higher risk rates of such incidents in mental health and community hospitals. While giving staff choice and flexibility over their shift patterns might lead to lower incident rates for violence against staff and disruptive behaviour, all benefits appear to be lost when wards run with 12+ hour shifts only. Nurse managers and those in charge of creating rotas for nursing staff should avoid implementing 12+ hour shifts as a blanket intervention for all staff. Further studies are needed to shed light on whether staff choice acts as a moderator between shift length and patient incidents.

## Figures and Tables

**Table 1:** Negative binomial and Poisson mixed-effects regression coefficients for the association between 12+ hour shifts and patient incidents.

Negative binomial mixed-effects regressions				
	Patient incidents (unadjusted)		Patient incidents (adjusted^‡^)	
	B	SE	B	SE

Proportion of long shifts	0.34^*∗*^	0.19	0.48^*∗*^	0.20
Proportion of long shifts squared	−1.02^*∗*^	0.54	−1.33^*∗*^	0.57
Proportion of long shifts cubic	0.73^*∗*^	0.40	1.08^*∗*^	0.43
	AIC	BIC	AIC	BIC
AIC/BIC	101212.7	101265.6	100938.8	101027.1

	Patient incidents with any harm (unadjusted)		Patient incidents with any harm (adjusted^‡^)	
	B	SE	B	SE

Proportion of long shifts	−0.09	0.30	0.01	0.32
Proportion of long shifts squared	0.76	0.86	0.54	0.95
Proportion of long shifts cubic	−0.47	0.64	−0.23	0.70
	AIC	BIC	AIC	BIC
	54659.9	54712.9	54609.8	54698.0

	Violence against staff (unadjusted)		Violence against staff (adjusted^‡^)	
	B	SE	B	SE

Proportion of long shifts	−0.33	0.41	−0.06	0.47
Proportion of long shifts squared	−2.38	1.32	−2.74	1.54
Proportion of long shifts cubic	2.17^*∗*^	0.95	2.63^*∗*^	1.12
	AIC	BIC	AIC	BIC
	32452.7	32505.7	32323.0	32411.3

	Self-injury (unadjusted)		Self-injury (adjusted^‡^)	
	B	SE	B	SE

Proportion of long shifts	0.77^*∗*^	0.45	0.83^*∗*^	0.48
Proportion of long shifts squared	−0.49	1.43	−0.66	1.54
Proportion of long shifts cubic	0.31	0.98	0.54	1.06
	AIC	BIC	AIC	BIC
	28105.2	28158.2	28007	28095.3

	Disruptive behaviour (unadjusted)		Disruptive behaviour (adjusted^‡^)	
	B	SE	B	SE

Proportion of long shifts	0.75	0.57	1.03^*∗*^	0.55
Proportion of long shifts squared	−4^*∗*^	1.82	−3.83^*∗*^	1.77
Proportion of long shifts cubic	3.09^*∗*^	1.24	3.27^*∗*^	1.22
	AIC	BIC	AIC	BIC
	23062	23115	22879.1	22967.4

Poisson mixed-effects regressions				
	Medication management incidents (unadjusted)		Medication management incidents (adjusted^‡^)	
	B	SE	B	SE

Proportion of long shifts	0.36	0.52	0.54	0.58
Proportion of long shifts squared	−0.59	1.51	−0.72	1.75
Proportion of long shifts cubic	0.12	1.22	0.13	1.41
	AIC	BIC	AIC	BIC
	21903.3	21947.5	21856.9	21936.3

	Falls (unadjusted)		Falls (adjusted^‡^)	
	B	SE	B	SE

Proportion of long shifts	0.23	0.55	0.19	0.56
Proportion of long shifts squared	−0.50	1.72	−0.63	1.72
Proportion of long shifts cubic	0.16	1.49	0.37	1.49
	AIC	BIC	AIC	BIC
	25211.8	25256.0	25207.4	25286.9

^*∗*^ *p* < 0.05. ^**‡**^Controlled for the number of nursing staff per bed, proportion of substantive shifts, skill-mix (registered nurses hours/registered nurses + healthcare support worker + nursing associate hours), and type of ward (physical vs. mental health).

## Data Availability

The data are available from the corresponding author upon request.

## Supplementary Materials

Supplementary Materials Figure S1 reports the use of long shifts at the ward level. Table S1 reports the full outputs of negative binomials mixed-effects and Poisson regressions for the association between 12+ hour shifts and patient incidents. Table S2 reports the outputs of negative binomial mixed-effects regressions for the association between 12+ hour shifts and patient incidents, sensitivity analyses excluding outlier ward. Figure S2 reports the sensitivity analysis, associations between the proportion of 12+ hour shifts and all incidents and violence against staff. All specific items of Supplementary Material have been referenced at appropriate points within the manuscript.
