# Supplementary material for: Nursing 12-Hour Shifts and Patient Incidents in Mental Health and Community Hospitals: A Longitudinal Study Using Routinely Collected Data
Source: J Nurs Manag. 2023 Sep 6;2023:6626585. doi: 10.1155/2023/6626585 (PMC11919144; doi:10.1155/2023/6626585)

Figure S1 Deployment of long shifts at the ward level

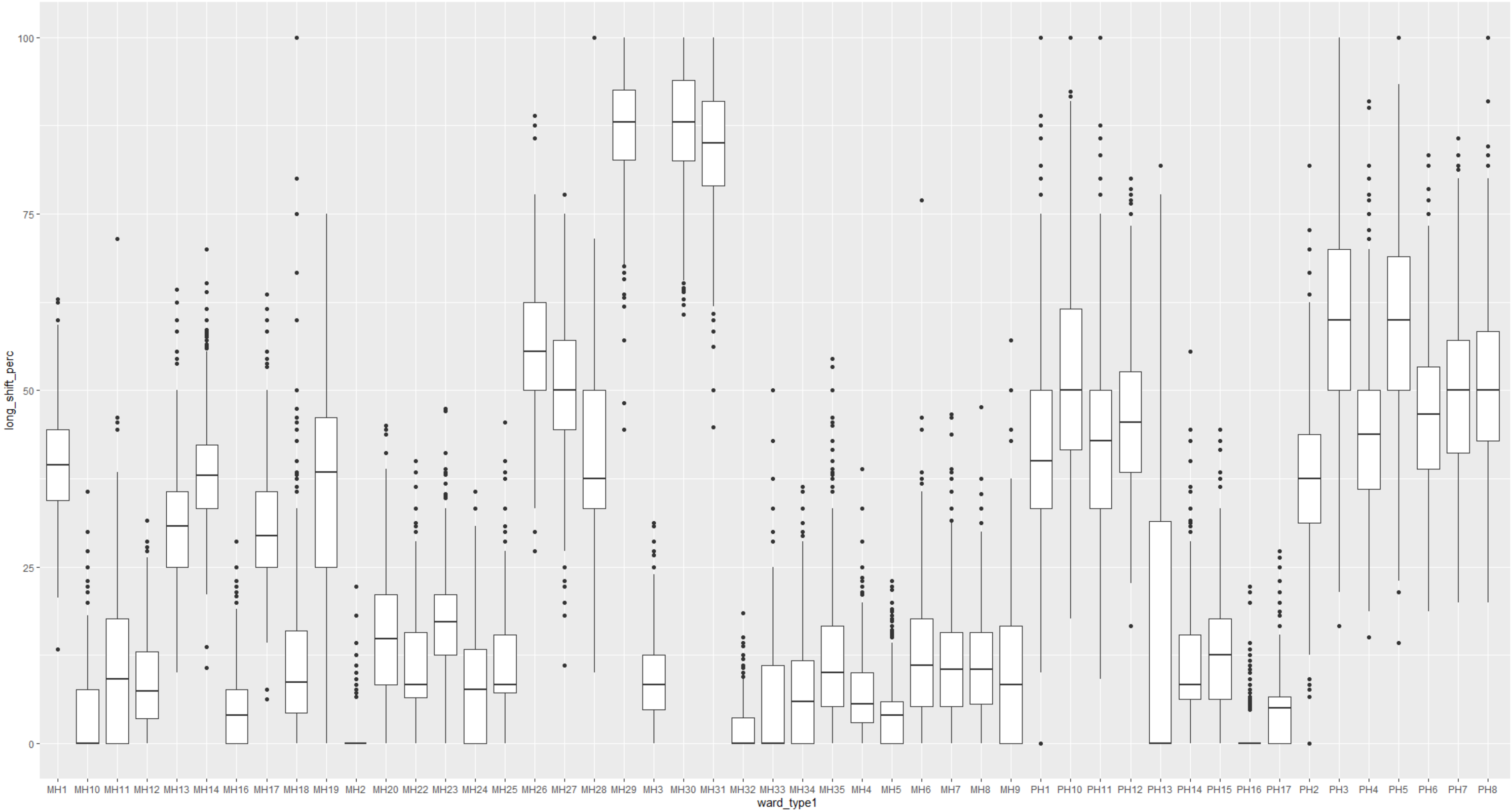

Table S1 Full outputs of negative binomials mixed-effects and Poisson regressions for the association between 12+ hours shifts and patient incidents

**Patient incidents**		
	**B**	**SE**
Intercept	-3.917*	0.20
Long shifts	0.485*	0.20
Long shift squared	-1.330*	0.57
Long shifts cubic	1.083*	0.43
Staff per bed	0.140*	0.01
Substantial shifts	-0.109*	0.05
Hours worked by RNs	0.113	0.09
Mental Health ward (ref: Physical Health)	0.696*	0.24
AIC	100938.8	
BIC	101027.1	
**Patient incidents with any harm**		
	**B**	**SE**
Intercept	-4.768*	0.26
Long shifts	0.01	0.32
Long shift squared	0.549	0.95
Long shifts cubic	-0.232	0.70
Staff per bed	0.107*	0.01
Substantial shifts	-0.131	0.08
Hours worked by RNs	0.050	0.15
Mental Health ward (ref: Physical Health)	0.403	0.29
AIC	54609.8	
BIC	54698.1	
**Violence against Staff**		
	**B**	**SE**
Intercept	-7.24*	0.32
Long shifts	-0.06	0.47
Long shift squared	-2.74	1.54
Long shifts cubic	2.63*	1.12
Staff per bed	0.16*	0.01
Substantial shifts	0.06	0.11
Hours worked by RNs	-0.56*	0.20
Mental Health ward (ref: Physical Health)	2.53*	0.36
AIC	32323.0	
BIC	32411.3	
**Fall**		
	**B**	**SE**
Intercept	-4.95*	0.33
Long shifts	0.19	0.55
Long shift squared	-0.63	1.72
Long shifts cubic	0.37	1.49

Staff per bed	0.05	0.03
Substantial shifts	-0.06	0.14
Hours worked by RNs	-0.02	0.24
Mental Health ward (ref: Physical Health)	-1.27*	0.36
AIC	25207.4	
BIC	25286.9	
**Self-Injury**		
	**B**	**SE**
Intercept	-11.05*	0.67
Long shifts	0.833	0.48
Long shift squared	-0.660	1.54
Long shifts cubic	0.547	1.05
Staff per bed	0.102*	0.01
Substantial shifts	-0.47*	0.11
Hours worked by RNs	0.34	0.20
Mental Health ward (ref: Physical Health)	5.46*	0.74
AIC	28007.0	
BIC	28095.3	
**Disruptive Behaviour**		
	**B**	**SE**
Intercept	-10.44*	0.45
Long shifts	1.028	0.55
Long shift squared	-3.835*	1.77
Long shifts cubic	3.274*	1.22
Staff per bed	0.185*	0.01
Substantial shifts	-0.057	0.13
Hours worked by RNs	1.015*	0.23
Mental Health ward (ref: Physical Health)	4.341*	0.48
AIC	22879.1	
BIC	22967.4	
**Medicines Misuse**		
	(β)	SE
Intercept	-6.46*	0.23
Long shifts	0.54	0.58
Long shift squared	-0.72	1.75
Long shifts cubic	0.13	1.41
Staff per bed	0.19*	0.02
Substantial shifts	0.48*	0.15
Hours worked by RNs	-0.31	0.26
Mental Health ward (ref: Physical Health)	0.41*	0.18
AIC	21856.9	
BIC	21936.3	

* $p < 0.05$

Table S2 Outputs of negative binomials mixed-effects regressions for the association between 12+ hours shifts and patient incidents – Sensitivity analyses excluding outlier ward

	**Patient incidents (adjusted‡)**	
	**B**	**SE**
Proportion of long shifts	0.75*	0.21
Proportion of long shift squared	-1.82*	0.61
Proportion of long shifts cubic	1.34*	0.45
	**AIC**	**BIC**
AIC/BIC	97242.6	97339.6
	**Patient incidents with any harm (adjusted‡)**	
	**B**	**SE**
Proportion of long shifts	0.24	0.33
Proportion of long shift squared	-0.04	0.95
Proportion of long shifts cubic	0.11	0.70
	**AIC**	**BIC**
	52500.4	52597.4
	**Violence against staff (adjusted‡)**	
	**B**	**SE**
Proportion of long shifts	0.52	0.50
Proportion of long shift squared	-3.18*	1.59
Proportion of long shifts cubic	2.73*	1.15
	**AIC**	**BIC**
	28953.8	29050.7

* p <0.05

‡Controlled for number of nursing staff per bed; proportion of substantive shifts; skill-mix (Registered Nurses hours/Registered Nurses + Healthcare Support Worker + Nursing Associate hours); type of ward (physical vs mental health)

Figure S2 Sensitivity analysis - associations between the proportion of 12+ hour shifts and all incidents and violence against staff

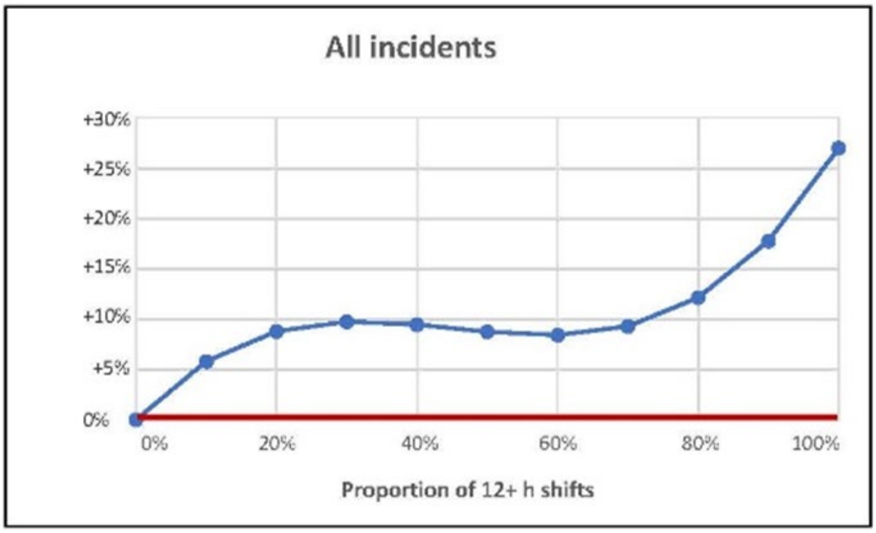

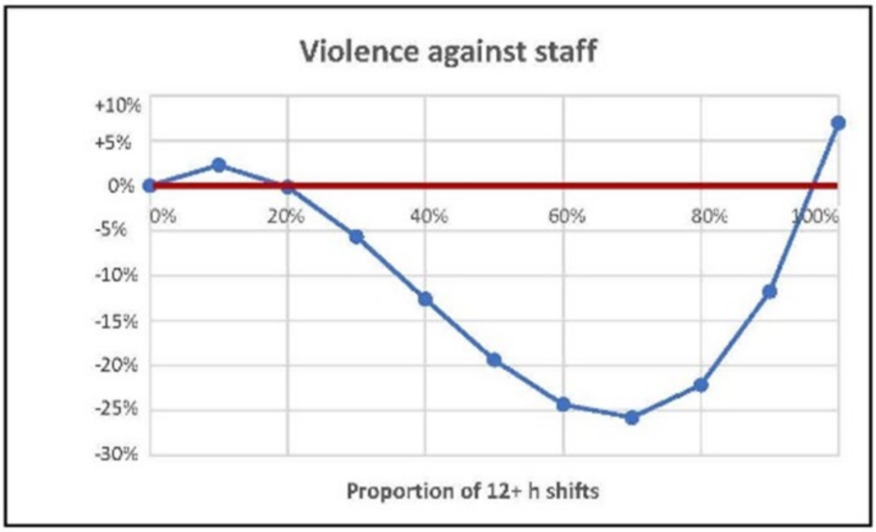

Supplement: Supplementary Materials — Figure S1 reports the use of long shifts at the ward level. Table S1 reports the full outputs of negative binomials mixed-effects and Poisson regressions for the association between 12+ hour shifts and patient incidents. Table S2 reports the outputs of negative binomial mixed-effects regressions for the association between 12+ hour shifts and patient incidents, sensitivity analyses excluding outlier ward. Figure S2 reports the sensitivity analysis, associations between the proportion of 12+ hour shifts and all incidents and violence against staff. All specific items of Supplementary Material have been referenced at appropriate points within the manuscript. [file 6626585.f1.pdf]
